# The Basis of Professional Secrecy

**Published:** 1903-12-12

**Authors:** 


### The Basis of Professional Secrecy.

The increased importance attached by the legislature and by public opinion to the survey and care of the public health has necessarily emphasised and multiplied the relationships existing between the medical profession and the various corporate bodies charged with the responsibilities of government in its several departments. Interesting as these relationships are in many of their aspects it still remains true that for the individual man or woman a question of more immediate concern is the position of the private practitioner towards those who seek guidance at his hands. Everyone allows that the official who deals with vital statistics and controls the municipal movements which regulate and preserve the health of the community is without question a functionary of great importance, but his concern appears to be not with individuals but with communities. In the public eye he is less a "doctor" than a municipal officer. His work and influence hardly seem to touch the personal associations of the individual citizen. With these, on the other hand, the general practitioner is most closely linked, and thus it remains true that in thinking or speaking of the profession the "plain man" exercises his judgment regarding those who carry the art of medicine into the homes of the citizens and to the bedside of the sick. The relationship thus established is a very intimate and confidential one, and it is unnecessary to enlarge upon its importance. At first sight it appears to be essentially a matter of private concern and to involve solely the welfare and happiness of the individual patient. As a matter of fact it rests upon a much broader basis. For surely it is in the public interest that every member of the community shall be encouraged to obtain expert advice whenever the condition of his health renders or appears to render this advisable. Any weakening of this claim opens the door to the opportunity for the spread of epidemic disease and tends to lessen the chance of restoring the sick and ailing to the ranks of efficient citizenship. It is, in a word, a matter of public concern that medical advice and skill shall be readily and promptly sought by those who need it. Now, whatever other conditions are necessary to secure this end, it is absolutely essential that the patient shall rely with unswerving confidence upon the secrecy of all communications he may make to his medical adviser. This claim may enjoy the sanction of professional custom and may be urged as an honourable obligation. But beyond or in addition to these it rests upon the secure basis of the general interest. Whatever weakens or minimises its force opposes that interest by establishing conditions which favour risk to the individual and danger to the public. If the sick man is to ask medical advice readily and to obtain its full value he must feel that frank speech implies a confidential reception. It is important to recognise this broad defence of professional secrecy, for there are influences at work which indicate how very far it is from receiving general recognition. Of course the question concerns the practitioner and still more the individual patient. But its true defence and justification are found on a wider outlook and in a recognition of the truth that anything which invades the private communications that pass between practitioner and patient is an offence against the common welfare. To this proposition, in its full proportions at least, it must be admitted that legal authorities refuse assent as is shown by the compulsion placed upon a witness in a Court of Law to reveal the secrets of the consulting room. And occasionally—an instance has occurred recently—police officials attempt to deal with members of the medical profession as if these latter were a branch of the detective force. Neither a medical practitioner nor any other citizen is justified in an attempt to hush up or conceal a crime of the evidence of which in an objective form comes under his attention. But it is no business of the medical profession to seek out crime by cross-examination of his patient or by other means. Unless the facts are conclusive of guilty and illegal action the presumption of a non-criminal explanation is justified. And, other reasons apart, it is justified, because in the last analysis it will be found that the confidence of the patient in the secrecy of his medical adviser, though perhaps a selfish instinct, is in entire harmony with the broader and commanding claim of public welfare.

